# Association of renalase with clinical outcomes in hospitalized patients with COVID-19

**DOI:** 10.1371/journal.pone.0264178

**Published:** 2022-03-08

**Authors:** Basmah Safdar, Melinda Wang, Xiaojia Guo, Charles Cha, Hyung J. Chun, Yanhong Deng, James Dziura, Joe M. El-Khoury, Fred Gorelick, Albert I. Ko, Alfred I. Lee, Robert Safirstein, Michael Simonov, Bin Zhou, Gary V. Desir

**Affiliations:** 1 Department of Emergency Medicine, Yale School of Medicine, New Haven, Connecticut, United States of America; 2 Department of Medicine, Yale School of Medicine, New Haven, Connecticut, United States of America; 3 Department of Cell Biology, Yale School of Medicine, New Haven, Connecticut, United States of America; 4 VA CT HealthCare, West Haven, Connecticut, United States of America; 5 Department of Surgery, Hartford HealthCare, Hartford, Connecticut, United States of America; 6 Yale Center of Analytics Sciences, New Haven, Connecticut, United States of America; 7 Department of Laboratory Medicine, Yale School of Medicine, New Haven, Connecticut, United States of America; 8 Department of Epidemiology of Microbial Diseases, Yale School of Public Health, New Haven, Connecticut, United States of America; University "Magna Graecia" of Catanzaro, ITALY

## Abstract

Renalase is a secreted flavoprotein with anti-inflammatory and pro-cell survival properties. COVID-19 is associated with disordered inflammation and apoptosis. We hypothesized that blood renalase levels would correspond to severe COVID-19 and survival. In this retrospective cohort study, clinicopathologic data and blood samples were collected from hospitalized COVID-19 subjects (March—June 2020) at a single institution tertiary hospital. Plasma renalase and cytokine levels were measured and clinical data abstracted from health records. Of 3,450 COVID-19 patients, 458 patients were enrolled. Patients were excluded if <18 years, or opted out of research. The primary composite outcome was intubation or death within 180 days. Secondary outcomes included mortality alone, intensive care unit admission, use of vasopressors, and CPR. Enrolled patients had mean age 64 years (SD±17), were 53% males, and 48% non-whites. Mean renalase levels was 14,108·4 ng/ml (SD±8,137 ng/ml). Compared to patients with high renalase, those with low renalase (< 8,922 ng/ml) were more likely to present with hypoxia, increased ICU admission (54% vs. 33%, p < 0.001), and cardiopulmonary resuscitation (10% vs. 4%, p = 0·023). In Cox proportional hazard model, every 1000 ng/ml increase in renalase decreased the risk of death or intubation by 5% (HR 0·95; 95% CI 0·91–0·98) and increased survival alone by 6% (HR 0·95; CI 0·90–0·98), after adjusting for socio-demographics, initial disease severity, comorbidities and inflammation. Patients with high renalase-low IL-6 levels had the best survival compared to other groups (p = 0·04). Renalase was independently associated with reduced intubation and mortality in hospitalized COVID-19 patients. Future studies should assess the pathophysiological relevance of renalase in COVID-19 disease.

## Introduction

As of December 2021, approximately 277 million global cases of COVID-19 had caused ~5.3 million deaths [1]. Disease manifestation ranges from asymptomatic to severe with extensive lung injury and death [2]. Approximately 81% of those infected have mild disease, 14% develop severe disease and 5% become critically ill with organ failure [3]. Critically ill patients with acute respiratory distress syndrome (ARDS) often require prolonged mechanical ventilation and have a mortality as high as 49% [4]. Thus, the importance of identifying host factors associated with severe COVID-19 infection to develop host-targeted therapeutics.

Although COVID-19 primarily manifests as a respiratory illness, there is substantial evidence of endothelial injury, thrombosis and dysfunction in vital organs including heart, kidney and brain. The systemic injury is mediated through the ACE2 receptor, a target for entry for SARS-COV2, which is highly expressed in pericytes of the endothelium and tissues of the same organs [5]. SARS-COV2 induces complement activation, and in severe cases, release of cytokines (IL-6, IL1β, IL-2, IL-18, IFNγ, TNFα, and VEGF). Cytokines decrease nitric oxide, resulting in loss of vascular integrity and multiorgan endothelial dysfunction [6–8]. Elevated IL-6 levels, in particular, is strongly associated with worse survival with COVID-19 [7]. Severe COVID-19 is also characterized by apoptosis of pericytes in the microvasculature [9], causing immune-thrombotic dysregulation, and development of inflammatory thrombi that lead to endothelial dysfunction and ARDS [10]. Recent evidence however questions the role of “COVID-19 cytokine storm”, since the inflammatory dysregulation in COVID-19 is neither as coordinated nor as intense as in other ARDS conditions [11, 12] Therefore, the quest to develop therapeutics that target cell death, endothelial dysfunction and immunomodulation in COVID-19 has high clinical relevance [13, 14].

Renalase (RNLS) is a recently discovered, highly conserved, ancient flavin adenine dinucleotide–dependent oxidase that metabolizes intracellular NADH [15–17]. Its secreted form promotes cell survival and downregulates the inflammatory response by signaling through the plasma-membrane calcium-ATPase ATP2B4, and activating growth and survival intracellular pathways (protein kinase B, JAK/STAT, and MAP kinase) [18]. In patients with acute renal, or cardiac injury low renalase has been associated with worse outcomes [19–22]. In animal models, renalase administration reduces cell injury through modulation of inflammation, preservation of vascular integrity via increase in nitric oxide, and preventing apoptosis [23–27]. In APOE knockout mice exposed to a high fat diet, tissue renalase levels inversely correlate with vascular plaque size [28], and increase with administration of the angiotensin 1 receptor antagonist Valsartan [28].

In light of renalase general cell survival properties, we studied a cohort of hospitalized COVID-19 patients to determine if its levels were independently associated with disease severity. Our findings reveal an inverse association between blood renalase levels and severe outcomes in COVID-19 patients, thus suggesting the utility of renalase levels in guiding disease management.

## Materials and methods

### Patient population

We conducted this study in hospitalized adult patients at Yale New Haven Hospital between March 1 and June 30, 2020, who had confirmed COVID-19. Among 3,450 eligible participants, baseline blood samples were available for 473 patients. Exclusions included patients <18 years, those who opted out of research on admission, or had insufficient samples. The protocol was approved by the Yale institutional review board (HIC 2000027792, 2000028383 and 2000027690). After abstraction of clinical data, data and samples were fully anonymized. Informed consent was required to have data/samples and medical records used for medical research.

### Study procedures

All patients had confirmed SARS-CoV-2 by RT-PCR of nasopharyngeal swab samples. All specimens and imaging were collected as part of routine medical care. Blood samples were collected by venipuncture and stored at -20°C.

### Patient profile and clinical data abstraction

Clinical data, including sociodemographics, comorbidities, vital signs, laboratory measurements, procedures, as well as disposition were extracted from the Department of Medicine COVID Explorer (DOM-CovX), a cohort of patients hospitalized with COVID [29]. We used Clarity, the relational enterprise database, which contains data from our hospital system’s instance of Epic (Verona, WI). A standard clinical protocol for treatments was implemented during the study period.

We conducted manual chart reviews to abstract admission date, presenting symptoms with date of onset, smoking history, immunocompromised status, cardiopulmonary resuscitation (CPR) and dates of intubation, death and last follow-up completed.

### Clinical outcomes

Primary composite outcome was defined as patient intubation or death within 180 days. Secondary outcomes included, a) mortality alone, b) use of intensive care unit (ICU), c) vasopressors, and d) cardiopulmonary resuscitations (CPR).

### Measurement of renalase and cytokines

Serum and plasma renalase levels were assayed as denaturation (acid)-sensitive pool by ELISA as described [30]. Inflammatory markers, including IFNγ, IFNλ, IFNα, IL-1β, IL-6, and TNFα, were measured using MSD V plex assays (Meso Scale Diagnostics, LLC, Rockville, MD), according to manufacturer’s instruction.

### Variable definitions

Baseline renalase was the first sample available for measurement after admission. Cut-off for the lowest quartile for renalase was 8,922 ng/ml and used to define low (n = 115) and high (n = 343) renalase levels. Disease severity on presentation was defined based on initial signs that have been shown to predict subsequent mortality. These included a) initial respiratory symptoms (defined as presence of shortness of breath, cough, chest pain or congestion), b) tachypnea (initial respiratory rate >24/min), c) initial hypoxia (<90% oxygen saturation), or d) initial hypotension (first systolic blood pressure of <90 mm Hg) [31, 32]. Time from initial symptom onset has also been shown to be a predictor of disease severity [2]. Progressive renal failure was determined based on estimated glomerular filtration rate using below 60 as abnormal. Markers of inflammation included high sensitivity C-reactive protein (hs-CRP), ferritin, procalcitonin, white blood cell count (WBC), albumin, TNF-α, IL-6, interferon-α2, IFN-λ, and IL-1. Markers of endothelial injury include troponin T, beta natriuretic peptide (BNP), D-dimer, and platelets.

### Statistical analysis

Data were summarized as percentage for categorical variables, mean and standard deviation (SD) for continuous variables. For univariate analyses, categorical variables between groups were compared using Fisher’s exact test or Chi-Square test and continuous variable were compared using student’s t test. All variables used had a missingness of <4%. No imputation was performed for missingness and analyses were conducted on complete cases. Correlation studies were conducted using Pearson correlation analysis. To evaluate the association of renalase and primary composite outcome, Kaplan-Meier curve was compared between renalase groups using log-rank test. Time to composite event was measured from the date of first measured renalase to the date of intubation or death (which ever happened first) and censored at the date of last follow-up. Kaplan-Meier analysis was also conducted on renalase groups categorized by using the cut off value derived from Youden index, which was estimated from the logistic regression model with renalase as predictor and composite event as outcome. Multivariate Cox proportional-hazards models were built in a hierarchical order, with renalase levels, laboratory measurements, demographics and confounders including metrics of disease severity added into the model sequentially. Clinically relevant confounders were selected a-priori based on univariate analysis findings, front line clinical experience and review of published data. Integrated time dependent AUC was reported for the final model. Similarly analytical approaches were also used for outcome of mortality. Kaplan-Meir curves were also generated when comparing renalase and IL-6 groups based on quartiles. Secondary outcomes were examined using unadjusted logistic regression. P-value <0.05 was considered statistically significant. All statistical analyses were performed using the statistical software SAS version 9.4 (Cary, NC).

### Patient and public involvement

Patients and the public were not involved in the research presented.

## Results

**Fig 1:**
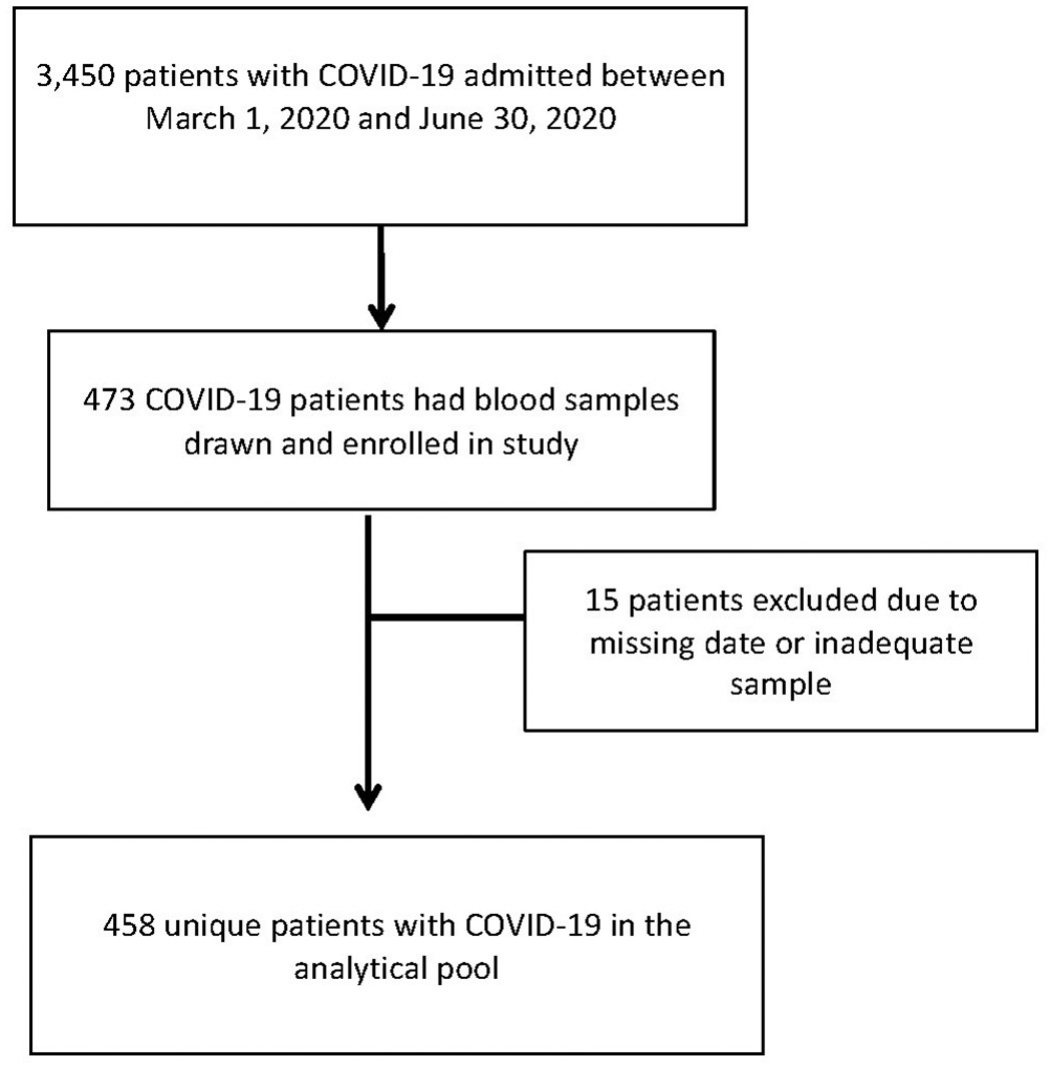
Flow chart of study patients from all COVID-19 admissions to the institution from March to July 2020.

Fig 1 shows 458 out of 473 patients formed the analytical sample. Study patients were similar in age and sex distribution but had higher comorbidities, and slightly longer hospital length of stay compared to the population of hospitalized patients admitted with COVID-19 between March-June 2020 (S1 Table in S1 File).

### Baseline characteristics

Table 1 shows patients had a mean age of 64 years (SD±17), with 53% males, 48% non-white, and 18% Hispanic. Mean renalase was 14,108 ng/ml (SD±8,137) and median 12,689 (range 803–69,829). Average length of admission was 16 days (SD±14) and average time to composite event was 62 days (SD±65).

**Table 1:** Baseline clinical profile of hospitalized COVID-19 patients with low and high renalase.

Factor	Total (n = 458)	High renalase (n = 343)	Low renalase (n = 115)	p-value
*Demographics*				
Age; mean (SD)	64·3 (17)	64·3 (17)	64·2 (17)	0·95
Male; n (%)	243 (53)	179 (52)	64 (56)	0·52
Hispanic; n (%)	83 (18)	62 (18)	21 (18)	0·96
Race; n (%)				0·34
White	239 (52)	177 (52)	62 (54)	
Black	140 (31)	111 (32)	29 (25)	
Other	79 (17)	55 (16)	24 (21)	
*Past Medical History*				
Hypertension; n (%)	315 (68)	234 (68)	81 (70)	0·81
Diabetes; n (%)	179 (40)	133 (40)	46 (41)	0·91
Hyperlipidemia; n (%)	178 (39)	133 (38)	45 (39)	0·89
Myocardial Infarction; n (%)	47 (11)	34 (10)	13 (12)	0·67
Congestive Heart Failure; n (%)	103 (23)	74 (22)	29 (26)	0·42
Chronic Pulmonary Disease	155 (35)	117 (35)	38 (34)	0·83
Chronic Kidney Disease; n (%)	99 (22)	75 (23)	24 (21)	0·82
Immunocompromised^a^; n (%)	73 (16)	46 (13)	27 (24)	0·008
Pregnancy; n (%)	6 (1)	5 (2)	1 (1)	0·64
Smoking; n (%)	181 (39)	129 (37)	52 (45)	0·13
*Symptoms at Presentation*				
Chest pain; n (%)	54 (12)	41 (12)	13 (11)	0·83
Cough; n (%)	306 (67)	233 (69)	73 (64)	0·38
Fever; n (%)	343 (76)	262 (77)	81 (71)	0·20
Dyspnea; n (%)	275 (61)	199 (59)	76 (67)	0·15
Gastrointestinal symptoms; n (%)	128 (28)	105 (31)	23 (20)	0·030
Days from symptom onset to sample drawn; mean (SD)	8 (7)	8 (7)	8 (8)	0·53
Days from presentation to sample drawn; mean (SD)	3 (6)	3 (5)	5 (7)	0·004
*Admission*				
BMI; mean (SD)	30·0 (8)	30·2 (8)	29·7 (7)	0·50
Initial Pulse; mean (SD)	95·8 (21)	96·0 (20)	95·2 (23)	0·75
Initial Systolic blood pressure; mean (SD)	135·2 (23)	136·3 (23)	131·7 (23)	0·07
Initial Diastolic blood pressure; mean (SD)	78·3 (16)	79·2 (16)	75·5 (14)	0·02
Initial O_2_ saturation; mean (SD)	94·2% (6)	94·8% (4)	92·4% (8)	0·004
Initial Hypoxia; n (%)	53 (12)	34 (10)	19 (16)	0·06
Initial Respiratory rate; mean (SD)	20·1 (7)	20·1 (7)	20·2 (5)	0·84
Initial Temperature; mean (SD)	99·5 (2)	99·6 (2)	99·5 (2)	0·65
Initial Laboratory Findings				
WBC; mean (SD)	7·3 (4)	7·1 (4)	7·9 (5)	0·08
Hemoglobin; mean (SD)	12·9 (2)	12·9 (2)	12·9 (2)	0·81
Platelet; mean (SD)	217·6 (93)	215·6 (90)	223·7 (104)	0·47
Creatinine; mean (SD)	1·5 (2)	1·4 (2)	1·5 (2)	0·89
eGFR; mean (SD)	50·1 (16)	50·4 (16)	49·3 (15)	0·54
Troponin; mean (SD)	44·0 (119)	45·3 (129)	39·1 (64)	0·55
INR; mean (SD)	1·0 (0·4)	1·0 (0·5)	1·0 (0·2)	0·95
D-dimer; mean (SD)	2·8 (6)	2·7 (6)	3·1 (7)	0·53
Ferritin; mean (SD)	980·5 (1633)	957·4 (1722)	1049·9 (1336)	0·56
Fibrinogen; mean (SD)	510·3 (145)	509·9 (144)	511·4 (146)	0·93
Procalcitonin; mean (SD)	0·6 (2)	0·4 (1)	1·2 (5)	0·07
hsCRP; mean (SD)^b^	90·8 (81)	90·3 (80)	93·5 (84)	0·69
Clinical Course				
Hospital length of stay; mean (SD)	16·2 (14)	15·0 (13)	20·0 (16)	0·003
ICU length of stay; mean (SD)	8·8 (10)	7·9 (10)	10·7 (12)	0·11
ICU Admission; n (%)	172 (38)	111 (33)	61 (54)	<0·001
Death free or discharged within 30 days; n (%)	230 (52)	182 (41)	48 (43)	0·043
Use of vasopressors; n (%)	96 (22)	57 (17)	39 (35)	<0·001
Hemodialysis; n (%)	31 (7)	21 (6)	10 (9)	0·34
CPR; n (%)	25 (5)	14 (4)	11 (10)	0·023
Discharge; n (%)				0·007
Home	271 (59)	214 (63)	57 (50)	
Nursing Facility	92 (20)	70 (21)	22 (19)	
Hospice / Expired	75 (16)	46 (14)	29 (25)	
Rehabilitation	12 (3)	7 (2)	5 (4)	
Other/Missing	12 (3)	9 (3)	3 (3)	

^**a**^ Immunocompromised = active cancer, HIV, liver disease, transplant (solid organ / bone marrow), leukemia, lymphoma, systemic lupus erythematous, and pregnancy

^b^hsCRP = high sensitivity CRP

Patients with low renalase were similar in age, sex, race, ethnicity compared to patients with high renalase. The two groups had similar common laboratory values and prevalence of comorbidities, such as hypertension, diabetes, chronic pulmonary disease, except for the higher proportion of immunocompromised patients observed in the low renalase group (24% vs. 13% p = 0·008). Average time from hospital admission to sample collection was 2 days longer in low renalase group compared to high renalase (3 days vs. 5 days; p = 0·004), although both groups were 8 days (SD±7 vs. SD±8) after onset of symptoms (Table 1).

### Renalase and primary composite outcome

Average time for follow up in our cohort was 77·6 days (SD±63). S2 Table in S1 File shows the characteristics associated with composite outcomes including male sex, hypoxia, elevated respiratory rate, history of congestive heart failure, and elevated WBC, troponin, D-dimer, and hs-CRP as well as low platelet count (p<0·01).

**Fig 2:**
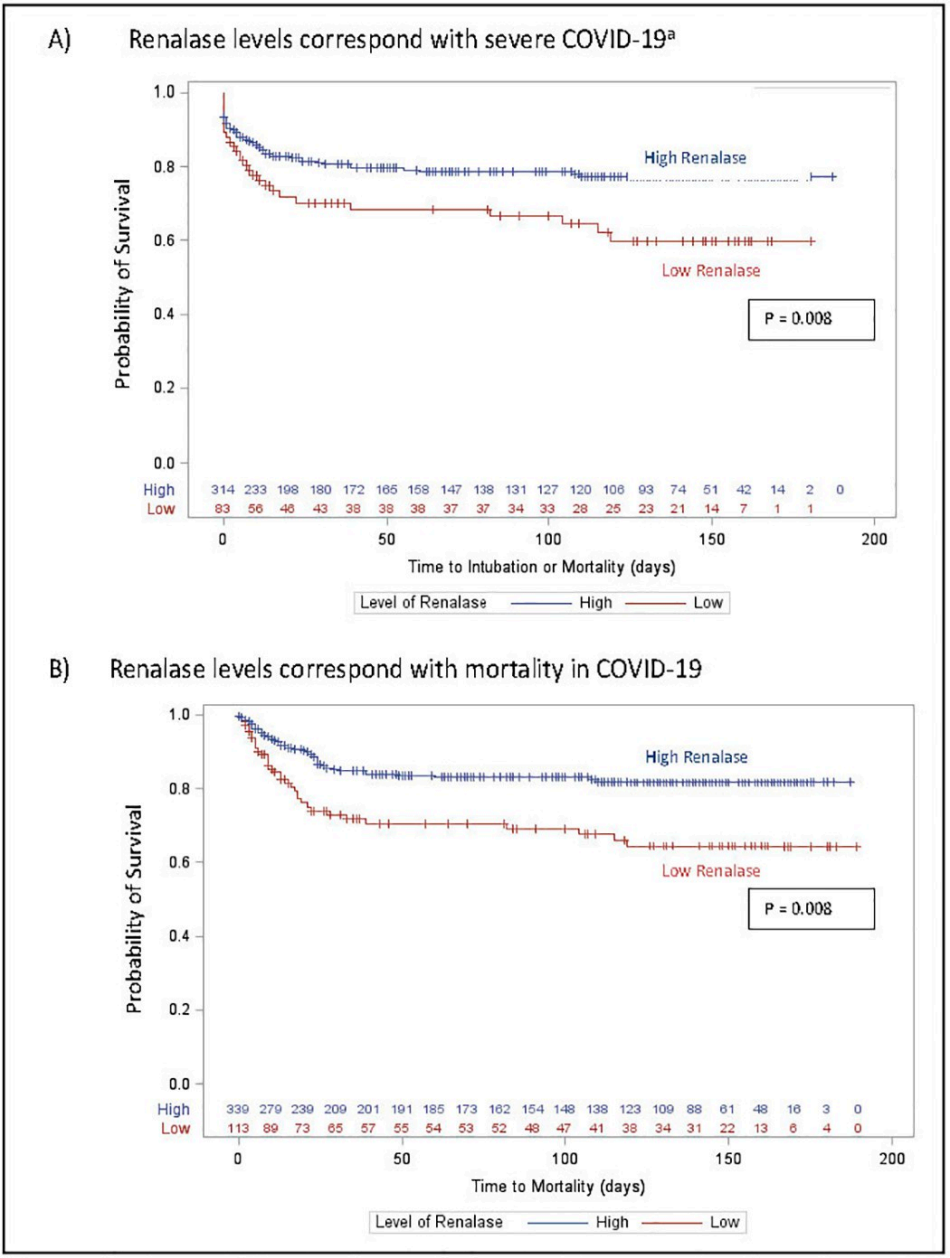
Time adjusted survival curves for high vs. low renalase among patients with COVID-19 disease (using first quartile as cutoff). Kaplan-Meir curves created and compared using log-rank test. (A) For composite outcome^a^: High renalase; Low renalase and (B) For Mortality: High renalase; Low renalase. ^a^ For composite outcomes, patients with renalase samples drawn after intubution were excluded from survival analysis (n = 55).

Fig 2 shows patients with high renalase had significantly longer time to primary composite event (Fig 2a: intubation or mortality) compared to patients with low renalase. Sensitivity analysis using the cuff off value derived from Youden index showed similar results (p<0·0001; S1 Fig in S1 File).

Sequential Cox proportional hazard models showed renalase used as a continuous measure was significantly associated with primary composite outcome—a) alone (HR 0·94; 95% CI 0·91–0·97); b) plus IL-6 (HR 0·94; 95% CI 0·91–0·97); c) plus socio-demographics (HR: 0·94; 95% CI 0·91–0·97); and d) plus comorbidities (Table 2). For every 1000 ng/ml increase in renalase, the risk of composite event decreased by 5% (hazard ratio = 0·95; CI 0·91–0·98), after adjusting for age, sex, BMI, race, comorbidities, inflammation, initial disease severity, and for time measurement from onset of symptoms and admission (Table 2).

**Table 2:** Cox hazard regression model of renalase and primary composite outcome. Model A of renalase for primary composite outcome; Model B of renalase, and IL-6, for primary composite outcome; Model C of renalase, IL-6, and demographic data for primary composite outcome; Model D of renalase, IL-6, demographic data, and additional confounders for primary composite outcome.

	Model A		Model B		Model C		Model D	
Variable	HR (95% CI)	p-value	HR (95% CI)	p-value	HR (95% CI)	p-value	HR (95% CI)	p-value
Renalase ng/ml (1000 units)	0·94 (0·91–0·97)	<0·001	0·94 (0·91–0·97)	<0·001	0·94 (0·90–0·97)	<0·001	0·95 (0·91–0·98)	<0·01
IL-6 (1000 units)			0·99 (0·96–1·03)	0·76	0·99 (0·96–1·03)	0·66	1·00 (0·97–1·03)	0·87
Age					1·02 (1·01–1·04)	<0·01	1·02 (1·00–1·04)	0·04
Male					1·54 (1·01–2·36)	0·04	1·29 (0·80–2·10)	0·30
Non-White					1·11 (0·71–1·73)	0·66	1·18 (0·70–2·00)	0·54
Disease Severity on Presentation^a^							0·98 (0·95–1·02)	0·40
BMI							0·86 (0·48–1·54)	0·60
Smoking History							0·99 (0·62–1·57)	0·96
Hypertension							1·27 (0·67–2·42)	0·47
Chronic Pulmonary Disease							0·77 (0·46–1·27)	0·30
High Sensitivity CRP							1·01 (1·00–1·01)	<0·001
Low estimated Glomerular Filtration							0·71 (0·43–1·19)	0·20
Myocardial Infarction							1·04 (0·54–2·01)	0·90
Time from admission to sample drawn (days)							0·93 (0·84–1·02)	0·11
Time from initial symptom to sample drawn (days)							0·94 (0·90–0·98)	<0·01
Immuno-compromised							0·97 (0·54–1·77)	0·93
***Time Integrated AUC Estimate***	***0***·***6362***		***0***·***6371***		***0***·***6178***		***0***·***7438***	

^a^ Derived Severity Indicator = Yes if patient had any of severity variables on presentation (Tachypnea, Hypoxia, Initial Hypotension and Initial Respiratory Symptoms)

The AUCs for each model are also summarized at the bottom of each Table and visually depicted with confidence intervals in S2 Fig in S1 File, indicating the stability of these models over time.

Fig 2b shows longer survival time in patients with high renalase compared to those with low renalase. S3 Table in S1 File showed for every 1000 ng/ml increase in renalase, survival improved by 5% (adjusted HR = 0·94; CI 0·90–0·98).

### Renalase and secondary outcomes

Patients with initial low renalase had significantly higher odds of, a) ICU admission (OR 2·28; 95% CI 1·48–3·51); b) use of vasopressors (OR 2·57; 95% CI 1·59–4·16); and c) cardiopulmonary resuscitation (OR 2·53; 95% CI 1·11–5·73). Mean renalase levels were lower in patients who needed vasopressors compared to those who did not (11925 versus 14705; p<0·001).

### Renalase and markers of endothelial injury and inflammation

**Fig 3:**
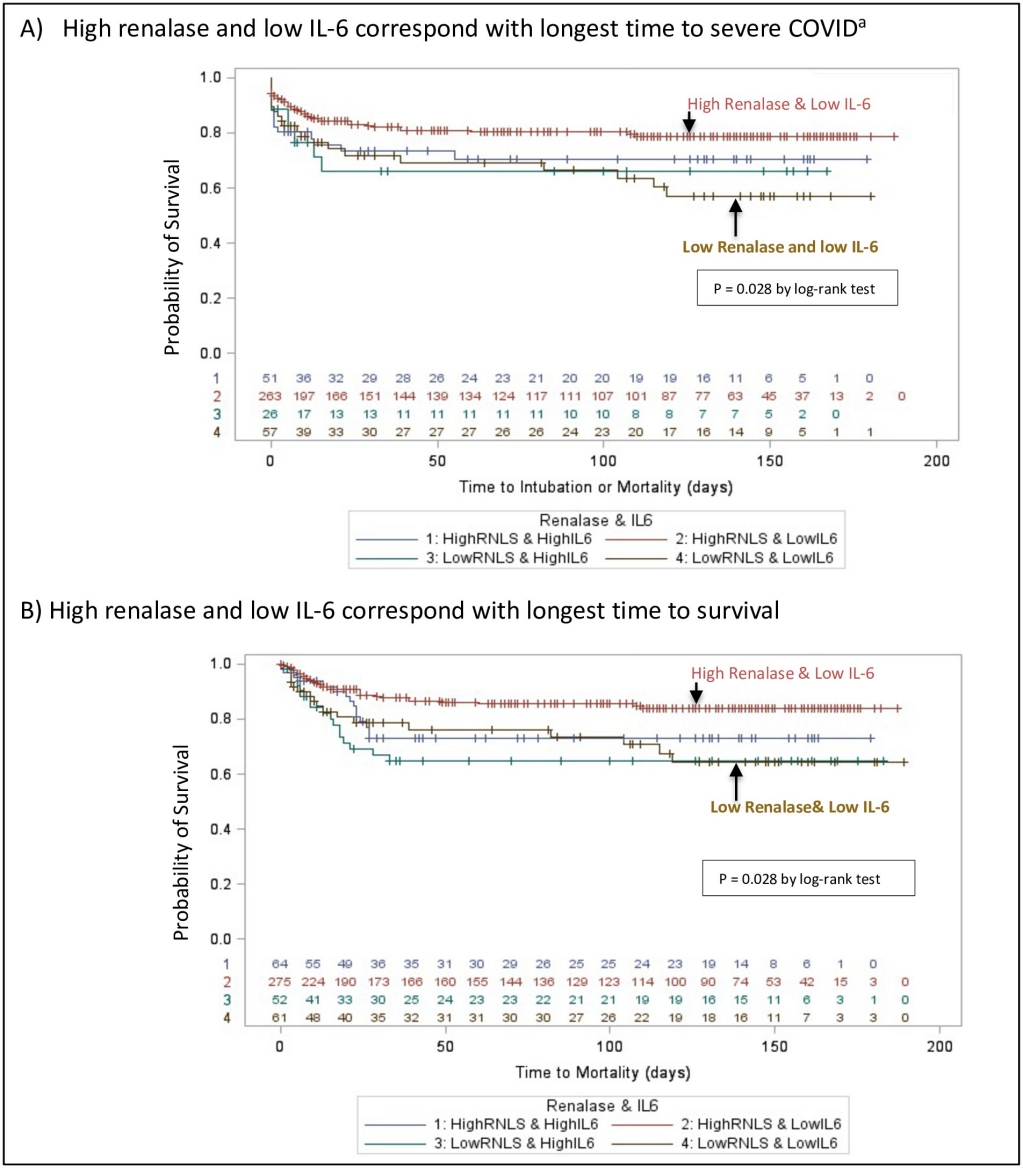
Time-adjusted survival curve for IL-6 and renalase among patients with COVID-19 disease (A) For composite outcome^a^: High renalase + High IL-6; High renalase + Low IL-6; Low renalase + High IL-6; Low renalase + Low IL-6 and (B) For Mortality: High renalase + High IL-6; High renalase + Low IL-6; Low renalase + High IL-6; Low renalase + Low IL-6. ^a^ For composite outcomes, patients with renalase samples drawn after intubution were excluded from survival analysis (n = 55).

We assessed the interaction effect of renalase and IL-6 on the primary outcome. Fig 3 shows patients with high renalase and low IL-6 had better survival, compared to other dyads. Of note, patients with low renalase and low-IL6 initially had an intermediate prognosis but eventually did as poorly as patients with low renalase-High IL-6. S2 Fig in S1 File shows renalase correlated with markers of cell injury (troponin), but not with markers of thromboembolism (D-dimer, platelets) (**S4 Table** in S1 File for distribution of endothelial injury and inflammation markers). Renalase also negatively correlated with markers of inflammation including ferritin, white blood cell count (WBC), and some cytokines (IL-1) (p<0·05).

## Discussion

In a large cohort of hospitalized COVID-19 patients, we report an association between severe COVID (mechanical ventilation or mortality) and blood renalase levels, after adjusting for known sociodemographic and clinical factors of severity. Compared to patients with high renalase levels, we found that those with low levels have worse COVID-19 disease outcomes, as defined by higher mortality as well as greater hypoxia, longer length of stay, higher ICU admission, use of vasopressors, and CPR rates. In addition, we noted that patients with high renalase and low IL-6 levels had significantly better survival than other quartiles. Finally, renalase did not correlate with thromboembolic proteins but did correlate with markers of cell injury and inflammation, suggesting a possible role of renalase in the vascular and inflammatory response / maladaptions in COVID-19 disease.

We report several findings that advance our knowledge of renalase in COVID-19. Renalase, a survival flavoprotein, is released in response to stress and protects cells and organs, including the endothelium, from stress injury. In this study, most patients with COVID-19 disease mounted a robust renalase response with values ranging up to 69,829 ng/ml. Comparatively, patients with a blunted response (low renalase group) had worse primary and secondary outcomes. Low renalase in similar range (8K ng/ml) has also been associated with adverse outcomes in acute pancreatitis in our prior work (data not published).

In our cohort, renalase was independently associated with survival, after adjusting for older age, presenting hypoxia, and well-known comorbidities. For every 1000 increase in renalase, survival improved by 5% and composite outcomes declined by 5%. This was noted in conjunction with IL-6, another well-established marker of mortality in COVID-19. Patients with high renalase-low IL6 had the best survival, while 61 patients in the low renalase-lowIL6 group did poorly, likely an indicator of ‘immune exhaustion’.

The survival benefit of renalase may be through its effect on inflammation and apoptosis. In our study, renalase was negatively correlated with ferritin [33] and WBC, both indicators of inflammatory response to dampen viral infection [33–35]. Renalase has also been shown to drive macrophage from M1-like macrophages (pro-inflammatory) to a more anti-inflammatory phenotype (M2-like macrophages), known to have a role in tissue repair/remodeling, thereby reducing ARDS severity [36, 37]. Similar to prior studies, we found patients have dysregulated cytokine responses to severe COVID-19 with elevations in some but not all inflammatory markers. The response differs from the coordinated cytokine storm seen in severe sepsis or in cancer patients [11]. Accordingly, we observed renalase was negatively correlated with IL-1 but not all inflammatory markers.

Apoptosis of endothelial cells has been noted in severe COVID-19 patients [9]. Viral entry is facilitated by endothelial pericytes, the multifunctional cells of the microvasculature that undergo apoptosis and have been implicated to have a direct role in microvascular dysfunction seen in COVID19. Renalase is also known to be expressed in the endothelial cells and has been shown to impede apoptosis. It has also been shown to correlate with a vascular adhesion protein 1, that modulates function of the endothelial pericytes. raising a possible protective antiapoptotic role of renalase on endothelial function [15, 38]. In our study, renalase correlated with markers of cell injury (troponin) but not with markers of thromboembolism (d-dimer, platelets). As an anti-inflammatory and anti-apoptotic survival factor, it is therefore possible renalase may play an immunomodulatory role [23, 25]; however, further studies are needed.

Renalase levels have been associated with plasma catecholamine levels rodent populations [39]. These studies suggest that renalase deficiency may be associated with increase in plasma catecholamine levels and increase in blood pressure. Although low RNLS levels in humans might also be associated with similar changes, thus improving hemodynamic stability, we find that despite this possibility, low RNLS levels are still significantly associated with worse overall clinical outcomes.

Our results should be interpreted in the context of its limitations. First, it was a single institution study from the ‘first wave’ in the US, which likely explains the high rates of intubation and mortality. However, the profile of our patients is similar to other cohorts with severe COVID-19 with comparable comorbidities and mortality, indicating the generalizability of our findings [40–42]. Second, our patient selection was based on availability of leftover blood samples drawn as part of clinical care. It is possible that patients with more severe COVID-19 disease may have had more blood drawn, affecting the availability of data provided in this study. However, we limited potential bias by restricting the analysis to the first blood sample available for the patient, excluding those who had an event before collection of blood sample and by comparing our cohort with the profile of all admitted patients at our hospital in the same time frame. Third, our results highlight the role of renalase in acute disease states where decreased in renalase level was associated with poor outcome. Similar dynamic relationship has been observed in other acute cardiac and renal diseases [20, 21, 27]. It should be noted that renalase may behave differently in type 1 diabetes [43] or in chronic inflammatory states such as systemic lupus erythematosus, where increased serum renalase has been observed in response to chronic inflammation and is attenuated with immunosuppressive therapy [44].

In summary, our results support the concept that sufficient plasma levels of renalase are critical for maintenance of biologic integrity and injury responses. Low renalase may be a cause and/or marker for the systemic biologic perturbations that lead to a poor outcome in COVID-19. It could also be useful to guide management and therapeutic interventions. Future studies should prospectively identify hospitalized patients with COVID-19 and matched controls and conduct time course studies to better understand the relationship between renalase and COVID-19 disease development. Additionally, the anti-inflammatory, anti-apoptotic and immunomodulatory role of renalase and its relationship with endothelial function should be explored further. Finally, the potential therapeutic value of renalase agonist administration, shown to effectively reduce acute disease severity in experimental models, should be examined in patients with severe COVID-19.

## Supporting information

S1 File (DOCX)
