# Supplementary material for: Association of renalase with clinical outcomes in hospitalized patients with COVID-19
Source: PLoS One. 2022 Mar 8;17(3):e0264178. doi: 10.1371/journal.pone.0264178 (PMC8903289; doi:10.1371/journal.pone.0264178)
Supplement: S1 File — (DOCX) [file pone.0264178.s001.docx]

**S1 Table. Clinical profile comparison between study cohort and non-enrolled hospitalized patients with COVID-19 from the same time period**

| **Factor** | **Study Cohort Hospitalized with COVID-19 (n=458)** | **Not enrolled Hospitalized with COVID-19 (n=2992)** | **p-value** |
| --- | --- | --- | --- |
| *Demographics* |  |  |  |
| Age; mean (SD) | 63·8 (18) | 63 (19) | 0·698 |
| Male; n (%) | 246 (53) | 1509 (51) | 0·272 |
| Hispanic; n (%) | 86 (19) | 809 (27) | <0·001 |
| Race; n (%)  White  Black  Other | 239 (52)  141 (31)  82 (18) | 1406 (47)  772 (26)  814 (27) | <0·001 |
| *Clinical Profile* |  |  |  |
| Hypertension; n (%) | 315 (68) | 1859 (62) | <0·001 |
| Diabetes; n (%) | 179 (40) | 1102 (37) | 0·202 |
| Hyperlipidemia; n (%) | 178 (39) | 1555 (52) | <0·001 |
| Myocardial Infarction; n (%) | 47 (11) | 351 (12) | 0·444 |
| Congestive Heart Failure; n (%) | 102 (23) | 647 (22) | 0·513 |
| Chronic Pulmonary Disease | 157 (35) | 598 (20) | <0·001 |
| Chronic Kidney Disease; n (%) | 99 (22) | 648 (22) | 0·833 |
| BMI; mean (SD) | 30·0 (8) | 29·6 (8) | 0·116 |
| *Clinical Course* |  |  |  |
| Hospital length of stay; mean (SD) | 16·2 (14) | 12·2 (14) | <0·001 |
| Discharge; n (%)  Home  Nursing Facility  Hospice / Expired  Rehabilitation  Other | 271 (59)  92 (20)  75 (16)  12 (3)  12 (3) | 1647 (56)  717 (24)  521 (17)  40 (1)  38 (1) | 0·058 |

**S2 Table. Baseline clinical profile of hospitalized COVID-19 patients with and without primary composite outcome**

| **Variable** | **Primary Composite Outcome** | | **p-value** |
| --- | --- | --- | --- |
|  | **Absent (n=308)** | **Present (n=144)** |  |
| *Demographics* |  |  |  |
| Age; mean (SD) | 63.4 (16) | 66.0 (18) | 0·13 |
| Male; n (%) | 147 (48) | 93 (65) | <0·001 |
| Hispanic; n (%) | 52 (17) | 31 (22) | 0·23 |
| Race; n (%)  White  Black  Other | 162 (53)  98 (32)  48 (15) | 74 (51)  39 (27)  31 (22) | 0·42 |
| *Past Medical History* |  |  |  |
| Hypertension; n (%) | 213 (69) | 101 (73) | 0·39 |
| Diabetes; n (%) | 122 (40) | 57 (41) | 0·74 |
| Hyperlipidemia; n (%) | 124 (40) | 53 (37) | 0·47 |
| Myocardial Infarction; n (%) | 31 (10) | 16 (12) | 0·63 |
| Congestive Heart Failure; n (%) | 60 (20) | 43 (31) | 0·007 |
| Chronic Pulmonary Disease | 115 (37) | 40 (29) | 0·09 |
| Chronic Kidney Disease; n (%) | 67 (22) | 32 (23) | 0·74 |
| Immunocompromised^a^ | 47 (15) | 24 (17) | 0·72 |
| Pregnancy; n (%) | 6 (2) | 0 (0) | 0·09 |
| Smoking; n (%) | 120 (39) | 60 (42) | 0·60 |
| *Symptoms at Presentation* |  |  |  |
| Chest pain; n (%) | 40 (13) | 13 (9) | 0·19 |
| Cough; n (%) | 209 (69) | 93 (65) | 0·38 |
| Fever; n (%) | 230 (76) | 109 (76) | 0·99 |
| Dyspnea; n (%) | 177 (59) | 95 (66) | 0·15 |
| Gastrointestinal symptoms; n (%) | 105 (35) | 22 (15) | <0·001 |
| Stage^b^  No symptoms  Stage I  Stage II  Stage III | 7 (2)  164 (53)  104 (34)  33 (11) | 1 (1)  78 (54)  38 (26)  27 (19) | 0·05 |
| Days from symptom onset to sample drawn; mean (SD) | 7·6 (6) | 8·8 (9) | 0·12 |
| Days from presentation to sample drawn; mean (SD) | 2·8 (5) | 4·3 (7) | 0·02 |
| *Admission* |  |  |  |
| BMI; mean (SD) | 30·1 (8) | 29·8 (8) | 0·69 |
| Pulse; mean (SD) | 93.9 (20) | 100·0 (22) | 0·004 |
| Systolic blood pressure; mean (SD) | 135·7 (24) | 133·9 (22) | 0·45 |
| Diastolic blood pressure; mean (SD) | 79·4 (15) | 75·9 (17) | 0·032 |
| Initial O_2_ saturation; mean (SD) | 95·5 (3) | 91·3 (8) | <0·001 |
|  |  |  |  |
| Respiratory rate; mean (SD) | 19·0 (4) | 22·7 (10) | <0·001 |
| Temperature; mean (SD) | 99·4 (2) | 99·9 (2) | 0·022 |
| *Initial Laboratory Findings* |  |  |  |
| WBC; mean (SD) | 6·9 (4) | 8·2 (5) | 0·006 |
| Hemoglobin; mean (SD) | 13·0 (2) | 12·9 (2) | 0·70 |
| Platelet; mean (SD) | 225·6 (92) | 199·8 (94) | 0·007 |
| Creatinine; mean (SD) | 1·4 (2) | 1·6 (3) | 0·27 |
| Initial Estimated  Glomerular Filtration; n (%) | 121 (39) | 65 (45) | 0.24 |
| eGFR; mean (SD) | 50·7 (15) | 48·8 (16) | 0·24 |
| Troponin; mean (SD) | 38·2 (134) | 57·1 (80) | 0.09 |
| INR; mean (SD) | 1·0 (0·3) | 1·1 (0·6) | 0·06 |
| D-dimer | 2·0 (4) | 4·4 (9) | 0·003 |
| Ferritin; mean (SD) | 828·0 (954) | 1318·8 (2537) | 0·029 |
| Fibrinogen; mean (SD) | 503·6 (138) | 525·1 (157) | 0·15 |
| Procalcitonin; mean (SD) | 0·3 (1) | 1·2 (4) | 0·012 |
| hsCRP; mean (SD)^c^ | 80·2 (74) | 114·5 (91) | <0·001 |
| *Clinical Course* |  |  |  |
| Hospital length of stay; mean (SD) | 13.2 (11) | 23.1 (17) | <0·001 |
| ICU length of stay; mean (SD) | 4·0 (4) | 12·2 (12) | <0·001 |
| ICU Admission; n (%) | 62 (20) | 110 (78) | <0·001 |
| Death and hospitalization free within 30 days; n (%) | 192 (63) | 38 (17) | <0·001 |
| Use of vasopressors; n (%) | 9 (3) | 87 (63) | <0·001 |
| Hemodialysis; n (%) | 16 (5) | 15 (11) | 0·029 |
| CPR; n (%) | 0 (0) | 25 (17) | <0·001 |
| Discharge; n (%)  Home  Nursing Facility  Hospice / Expired  Rehabilitation  Other | 235 (76)  65 (21)  3 (1)  2 (0·6)  4 (1) | 36 (25)  27 (19)  72 (50)  10 (7)  0 (0) | <0·001 |
| *Disease Severity* |  |  |  |
| Tachypnea; n (%) | 16 (5) | 27 (19) | <0·001 |
| Hypoxia; n (%) | 13 (4) | 40 (29) | <0·001 |
| Initial Hypertension; n (%) | 5 (2) | 7 (5) | 0·046 |
| Initial Respiratory Symptoms; n (%) | 252 (83) | 118 (82) | 0·80 |
| Disease Severity^d^ | 255 (83) | 123 (85) | 0·48 |

^a^ Immunocompromised = active cancer, HIV, liver disease, transplant (solid organ / bone marrow), leukemia, lymphoma, systemic lupus erythematous, and pregnancy

^b^ Stage I = 0-4·9 days; Stage II = 4-9·9 days; Stage III ≥ 10 days

^c^ hsCRP = high sensitivity CRP

^d^ Disease Severity=Yes if any of severity variables is Yes (Tachypnea, Hypoxia, Initial Hypertension and Initial Respiratory Symptoms)

**S3 Table. Cox hazard regression model of renalase and mortality·** Model A of renalase for mortality; Model B of renalase, and IL-6, for mortality; Model C of renalase, IL-6, and demographic data for mortality; Model D of renalase, IL-6, demographic data, and additional confounders for mortality

|  | **Model A** | | **Model B** | | **Model C** | | **Model D** | |
| --- | --- | --- | --- | --- | --- | --- | --- | --- |
| **Variable** | HR (95% CI) | p-value | HR (95% CI) | p-value | HR (95% CI) | p-value | HR (95% CI) | p-value |
| RNLS ng/ml (1000 units) | 0·94 (0·91-0·97) | <0·001 | 0·94 (0·90-0·97) | <0·001 | 0·93 (0·90-0·97) | <0·001 | 0·94 (0·90-0·98) | 0·001 |
| IL-6 (1000 units) |  |  | 0·94 (0·85-1·05) | 0·28 | 0·95 (0·86-1·05) | 0·34 | 0·96 (0·87-1·06) | 0·41 |
| Age |  |  |  |  | 1·05 (1·03-1·06) | <·0·001 | 1·05 (1·03-1·08) | <0·001 |
| Male |  |  |  |  | 1·42 (0·92-2·21) | 0·12 | 1·31 (0·80-2·13) | 0·29 |
| Non-White |  |  |  |  | 0·95 (0·59-1·54) | 0·84 | 0·90 (0·58-1·71) | 0·98 |
| BMI |  |  |  |  |  |  | 0·99 (0·95-1·03) | 0·57 |
| Disease Severity on presentation |  |  |  |  |  |  | 0·77 (0·43-1·39) | 0·39 |
| Smoking History |  |  |  |  |  |  | 1·29 (0·63-2·64) | 0·48 |
| Hypertension |  |  |  |  |  |  | 0·74 (0·44-1·25) | 0·26 |
| Chronic Pulmonary Disease |  |  |  |  |  |  | 1·00 (1·00-1·01) | 0·15 |
| High Sensitivity CRP |  |  |  |  |  |  | 0·80 (0·49-1·32) | 0·39 |
| Low estimated Glomerular Filtration |  |  |  |  |  |  | 1·33 (0·69-2·55) | 0·40 |
| Myocardial Infarction |  |  |  |  |  |  | 1·02 (0·99-1·06) | 0·28 |
| Time from admission to sample drawn (days) |  |  |  |  |  |  | 0·98 (0·95-1·02) | 0·44 |
| Time from initial symptom to sample drawn (days) |  |  |  |  |  |  | 1·44 (0·81-2·55) | 0·22 |
| Immuno-compromised |  |  |  |  |  |  | 0·94 (0·90-0·98) | <0·001 |
| ***Time Integrated AUC Estimate*** | ***0***·***6393*** | | ***0***·***6432*** | | ***0***·***7432*** | | ***0***·***7714*** | |

**S4 Table. Distribution of Endothelial and Inflammatory Markers**

| **Variable** | **1st Pctl** | **25th Pctl** | **50th Pctl** | **Mean** | **75th Pctl** | **99th Pctl** | **Std Dev** | **Range** |
| --- | --- | --- | --- | --- | --- | --- | --- | --- |
| Renalase | 1620·87 | 8933·08 | 12689·06 | 14108·43 | 17316·36 | 46427·61 | 8136·56 | 69026·65 |
| D-dimer | 0·18 | 0·58 | 0·93 | 2·76 | 1·85 | 33·89 | 6·05 | 35·03 |
| Platelet | 75·00 | 155·00 | 198·00 | 217·71 | 259·00 | 528·00 | 93·27 | 618·00 |
| Troponin | 0·01 | 0·01 | 0·01 | 0·03 | 0·01 | 0·42 | 0·11 | 1·77 |
| hsCRP | 1·00 | 23·60 | 70·60 | 90·83 | 135·60 | 300·00 | 80·71 | 299·40 |
| Ferritin | 22·00 | 264·00 | 602·00 | 980·56 | 1130·00 | 7229·00 | 1630·24 | 25094·00 |
| Procalcitonin | 0·06 | 0·06 | 0·12 | 0·60 | 0·26 | 11·50 | 2·44 | 42·14 |
| TNFα | 0·75 | 1·96 | 2·90 | 3·69 | 4·57 | 13·35 | 2·65 | 20·40 |
| IL-6 | 0·83 | 7·48 | 33·47 | 1075·66 | 394·40 | 17238·23 | 5814·88 | 104672·27 |
| IFN2α2 | 0·00 | 0·68 | 4·99 | 54·78 | 32·87 | 707·65 | 163·92 | 2327·50 |
| IFN λ | 0·00 | 21·05 | 60·33 | 100·62 | 127·50 | 554·32 | 123·68 | 879·70 |
| IFN | 0·86 | 15·33 | 61·80 | 380·90 | 187·97 | 6501·87 | 1343·32 | 17609·11 |
| WBC | 1·65 | 4·70 | 6·40 | 7·27 | 8·70 | 24·62 | 4·04 | 32·30 |
| IL-1 | 0·00 | 0·03 | 0·08 | 0·21 | 0·18 | 1·99 | 0·92 | 16·28 |

**S1 Fig. Sensitivity analysis showing time adjusted survival curves for high vs· low renalase among patients with COVID-19 disease (using Youden-index to identify cut off)·** Kaplan-Meir curves created and compared using log-rank test· (A) For composite outcome^a^: High renalase; Low renalase and (B) For Mortality: High renalase; Low renalase

| 1. Renalase Levels correspond with severe COVID-19^a^ | B. Renalase Levels correspond with Mortality in COVID-19^b^ |
| --- | --- |
| 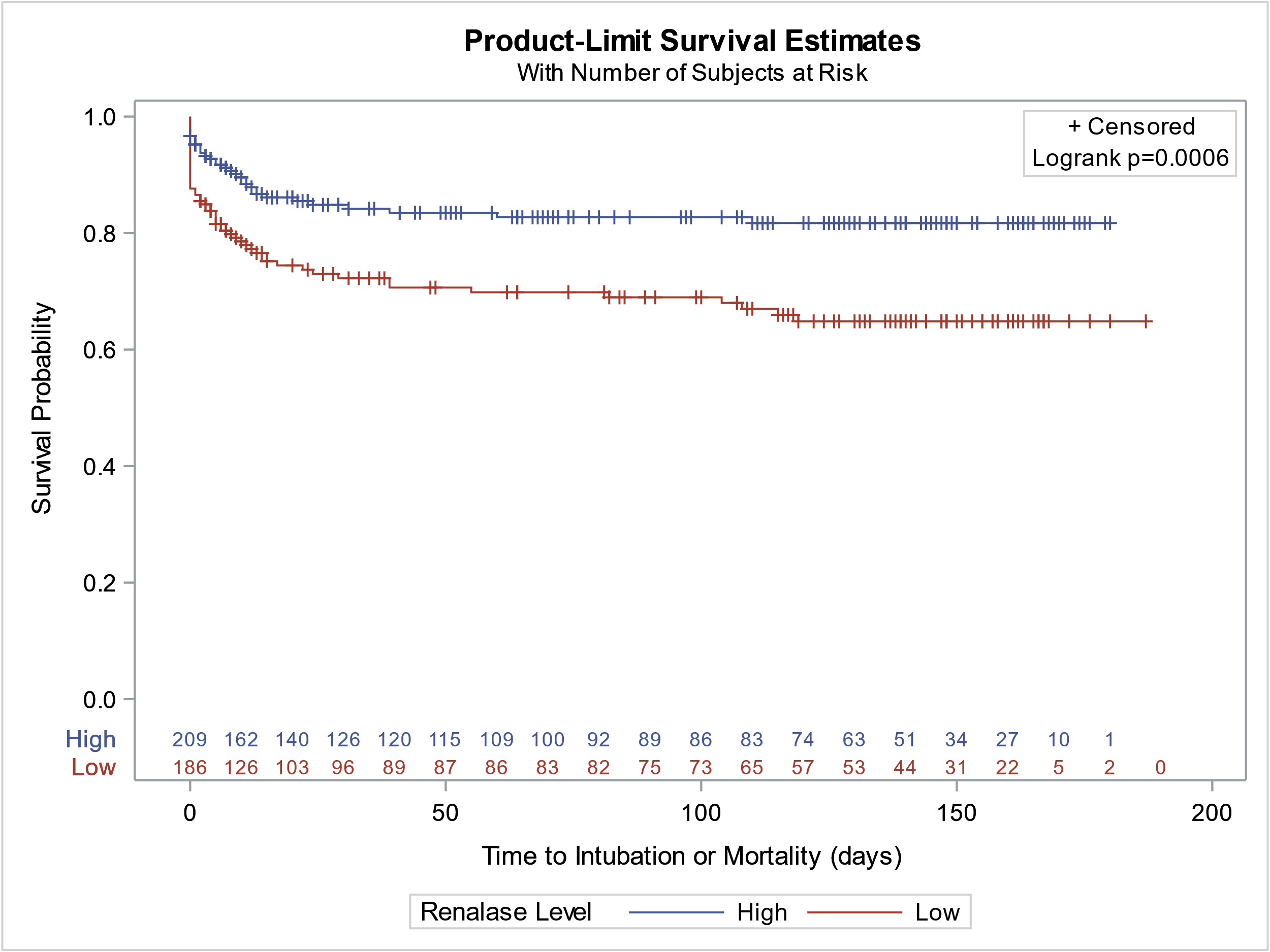 | 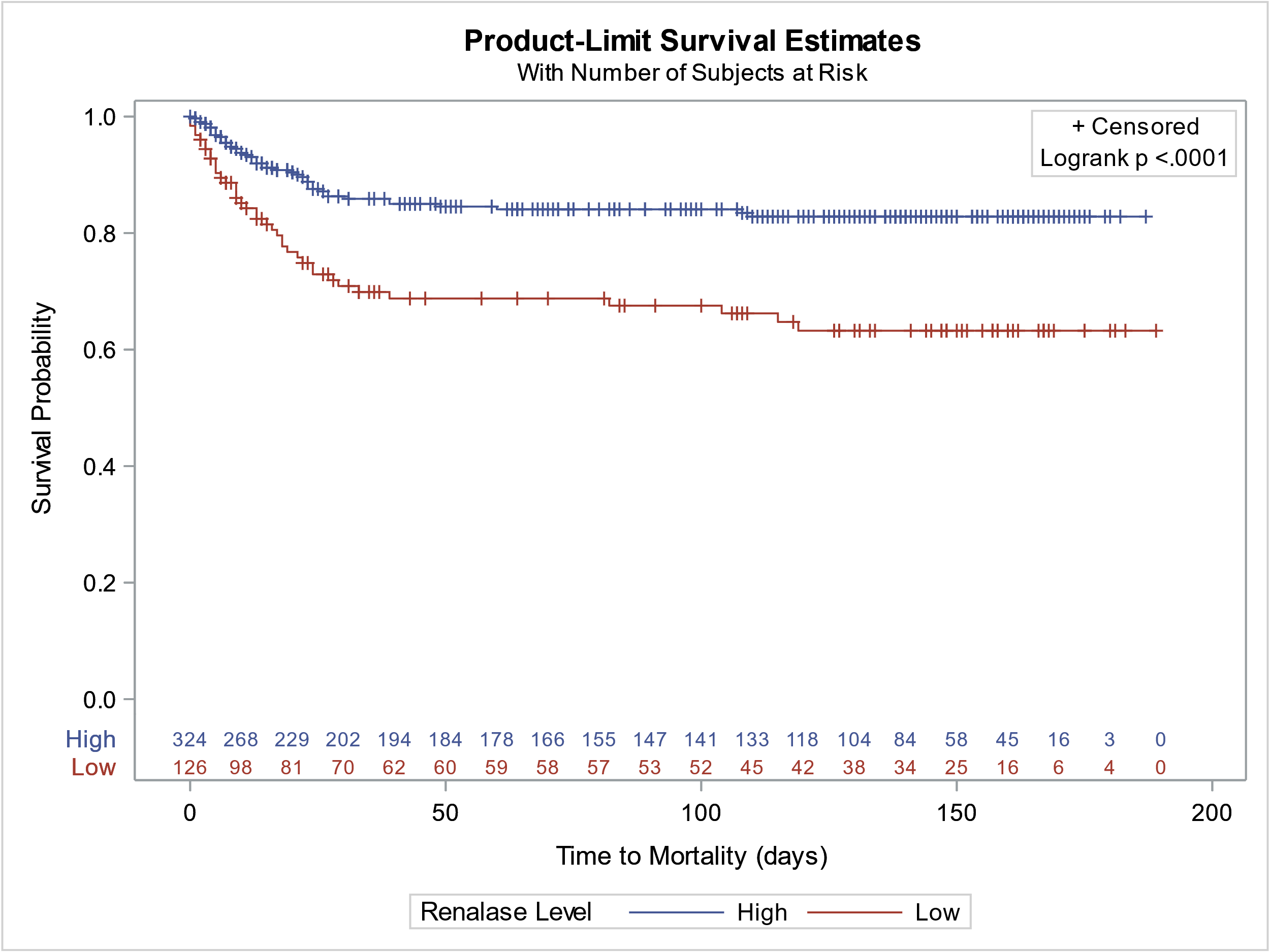 |

**S2 Fig. AUC/ROC curves for time to intubation or mortality and time to mortality using Model D.** (A) For composite outcomes and (B) For mortality outcomes

| 1. AUC/ROC Curves corresponding with Composite Outcome | B. AUC/ROC Curves corresponding with Mortality Outcome |
| --- | --- |
| 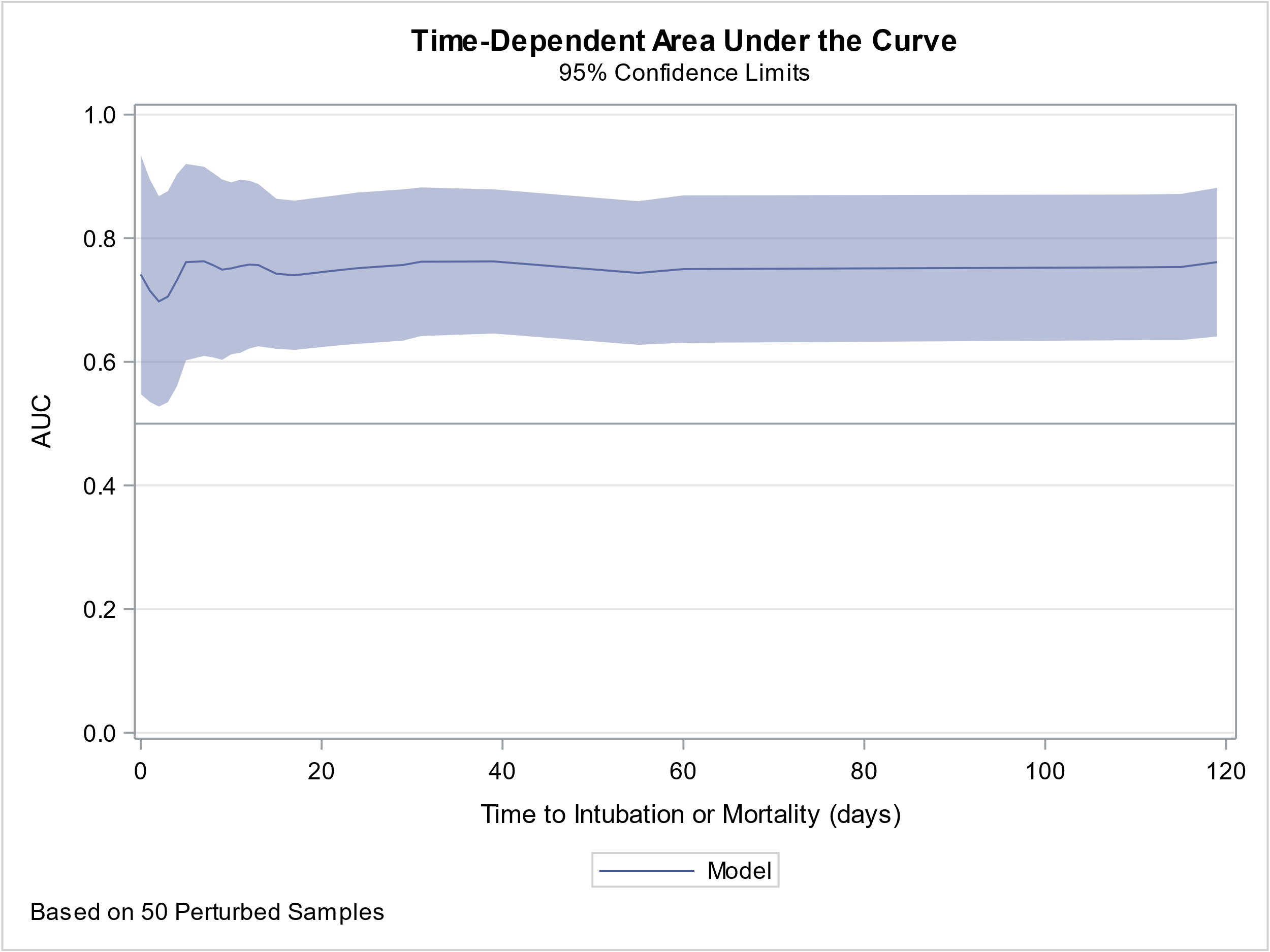 | 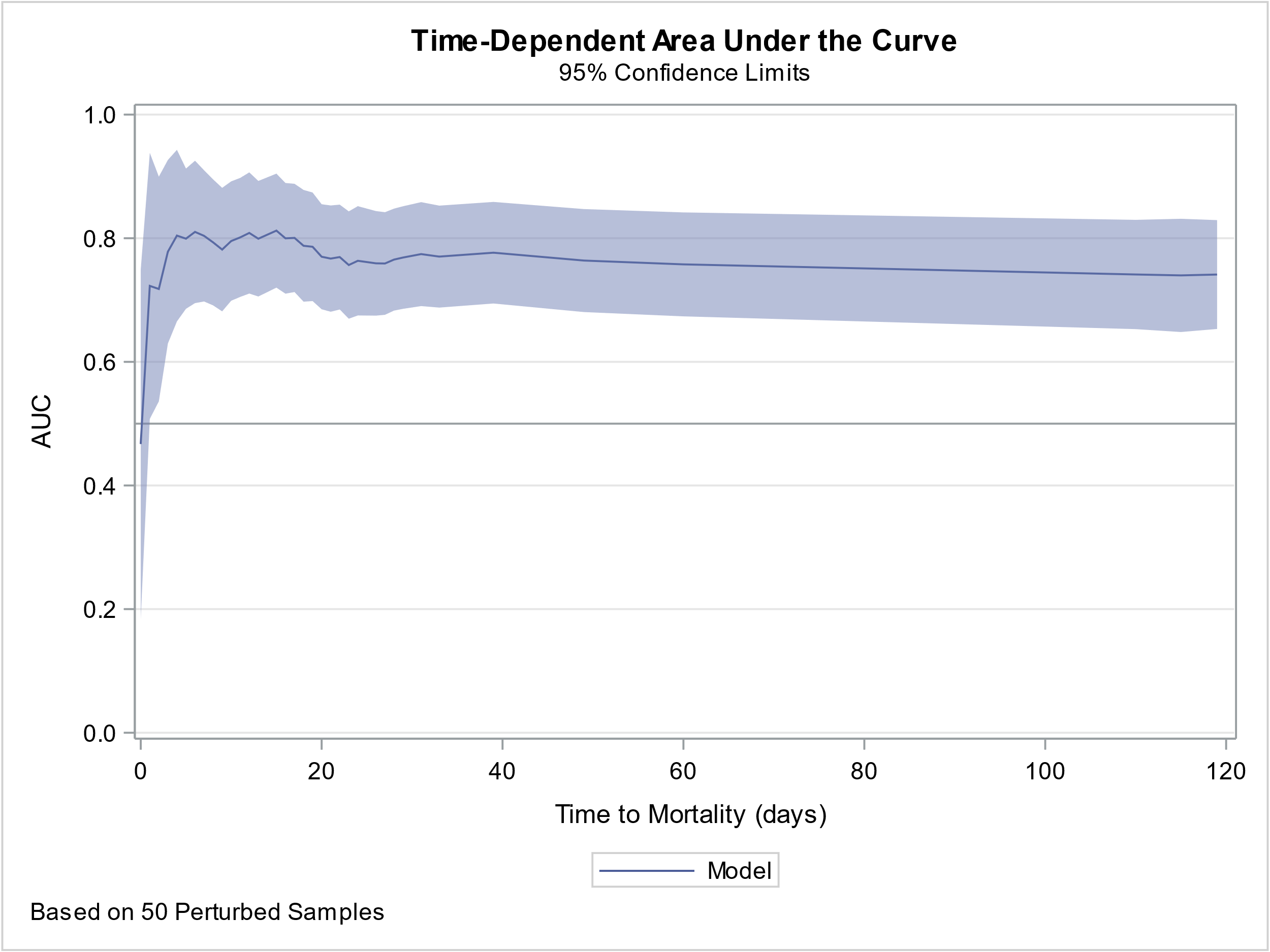 |
| 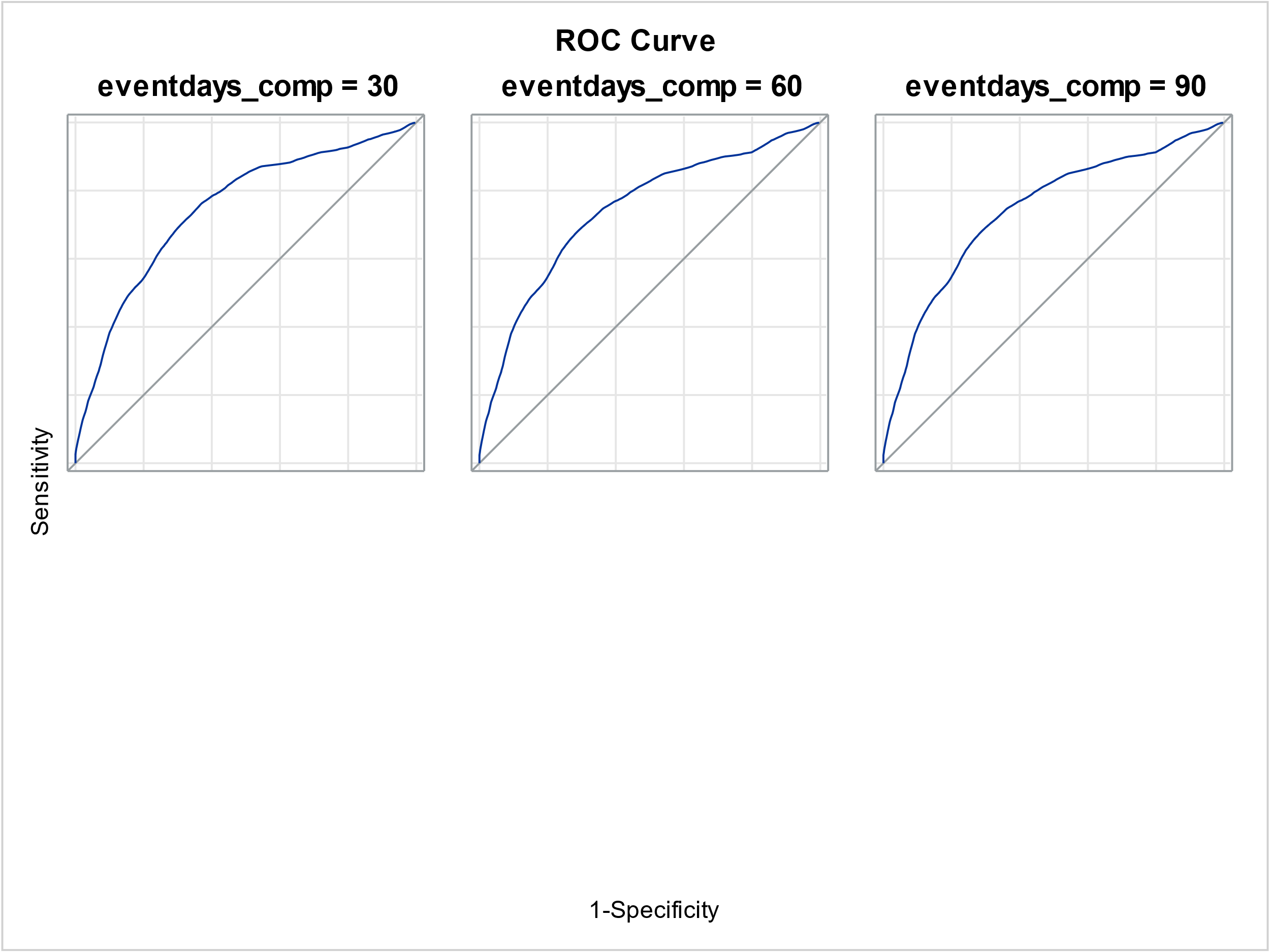 | 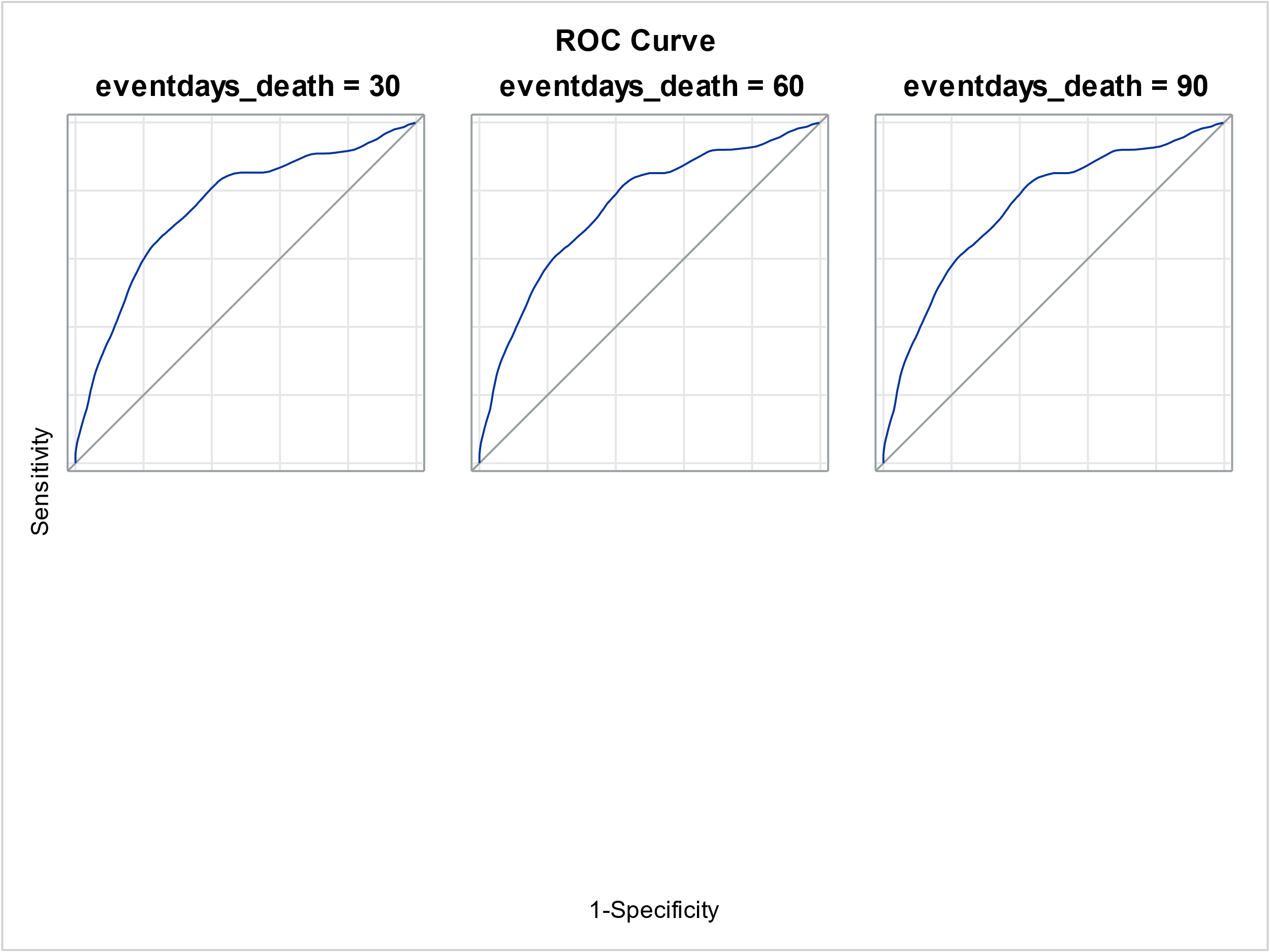 |

**S3 Fig. Correlation matrix of renalase with markers** of A) endothelial damage (D-dimer, platelet, troponin) and B) inflammation (high sensitivity CRP (hsCRP), ferritin, procalcitonin, WBC, TNFα, IL-6, IFN, IFN2α2, IFN-λ, and IL-1)**·**

**
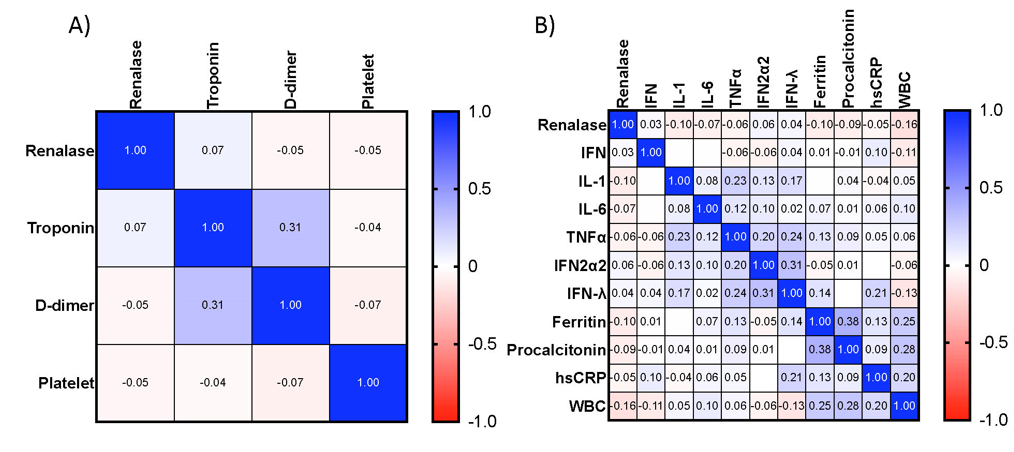
**
